# Advanced computational model of rod ERG kinetics

**DOI:** 10.1007/s10633-024-09977-8

**Published:** 2024-07-03

**Authors:** Christopher W. Tyler

**Affiliations:** 1 Smith-Kettlewell Brain Imaging Center, Smith-Kettlewell Eye Research Institute, 2318 Fillmore Street, San Francisco, CA 94115 USA; 2 Department of Optometry and Vision Sciences, School of Health Sciencesm University of London, Northampton Square, London, EC1V 0HB UK

**Keywords:** ERG, Computational model, Human, Flash intensity series, Photoreceptor potential, Bipolar response

## Abstract

**Purpose:**

The electroretinogram (ERG) is the summed response from all levels of the retinal processing of light, and exhibits several profound nonlinearities in the underlying processing pathways. Accurate computational models of the ERG are important, both for understanding the multifold processes of light transduction to ecologically useful signals by the retina, and for their diagnostic capabilities for the identification and characterization of retinal disease mechanisms. There are, however, very few computational models of the ERG waveform, and none that account for the full extent of its features over time.

**Methods:**

This study takes the neuroanalytic approach to modeling the ERG waveform, defined as a computational model based on the main features of the transmitter kinetics of the retinal neurons.

**Results:**

The present neuroanalytic model of the human rod ERG is elaborated from the same general principles as that of Hood and Birch (Vis Neurosci 8(2):107–126, 1992), but incorporates the more recent understanding of the early nonlinear stages of ERG generation by Robson and Frishman (Prog Retinal Eye Res 39:1–22, 2014). As a result, it provides a substantially better match than previous models of rod responses in six different waveform features of the ERG flash intensity series on which the Hood and Birch model was based.

**Conclusion:**

The neuroanalytic approach extends previous models of the component waves of the ERG, and can be structured to provide an accurate characterization of the full timecourse of the ERG waveform. The approach thus holds promise for advancing the theoretical understanding of the retinal kinetics of the light response.

## Introduction

The ERG is a powerful non-invasive assay of the functional integrity of the human retina, providing measures of the retinal receptor potential and the bipolar cell function that are the main topic of the present paper, together with signals attributable to the inner plexiform layer of the retina. In order to gain maximal information about the underlying retinal functions, however, it is important to have a comprehensive model of the ERG dynamics that can quantify the detailed changes in their properties over the full timecourse of the response, beyond the basic measures of the amplitudes and peak times of the a-wave (initial negative peak) and b-wave (main positive peak). The approach taken here is neuroanalytic modeling based on known properties of the underlying neural circuitry, rather than purely mathematical components. (It does not, however, attempt to characterize all aspects of the underlying biophysical processes, only the main features that are relevant to the characterization of the resultant ERG signal.)

**Fig. 1:**
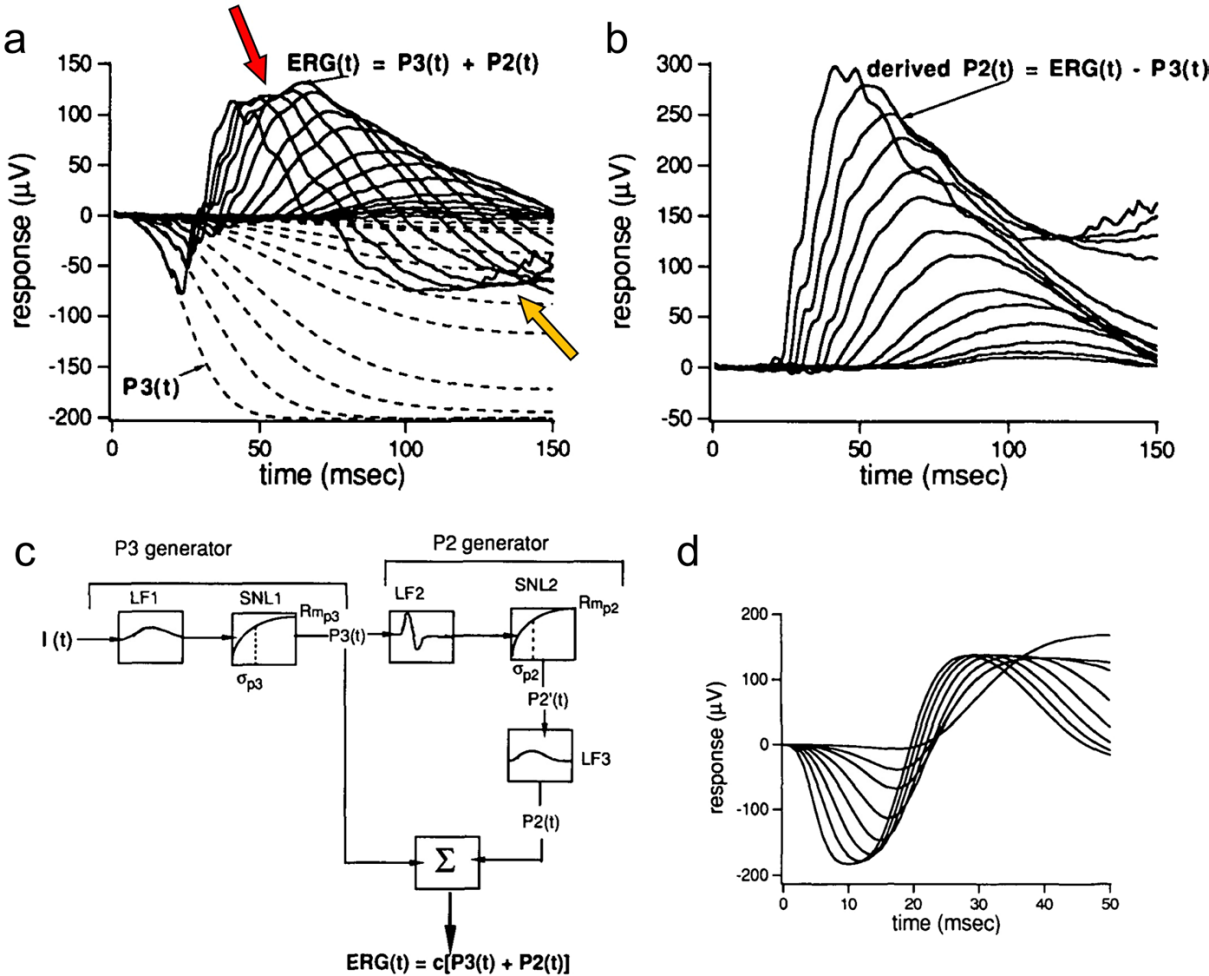
**a**, **b** Empirical derivation of the ERG P2 component underlying the b-wave for a set of dark-adapted ERG flash responses using flash intensities up to a maximum of 2.0 log scot td-s in ~ 0.2 log unit steps (from [6], Fig. 6; method of [4]). **c, d** Theoretical model of the a/b-wave complex (from [6], Figs. 8 and 9). See text for details

A relatively complete model of dark-adapted rod ERG kinetics as a function of flash intensity was developed by Hood and Birch [6, 7], whose data for the dark-adapted flash response as a function of intensity are depicted as the solid curves in Fig. 1a (see Methods for recording conditions). Both the initial negative a-wave and the subsequent positive b-wave increase gradually in amplitude and have progressively decreasing peak times with intensity, with the b-wave amplitude tending to saturate at the higher intensities. It is noteworthy that the data cross over the baseline to become negative at long durations, and even more negative than the a-wave minima for the lower flash intensities.

An initial approach to neuroanalytic modeling of these ERG functions by Hood and Birch [6], based on the analysis of Penn and Hagins [9] is depicted in Fig. 1, where they fitted the initial a-wave portion of their stack of flash ERG responses (solid curves in Fig. 1a) with a set of model responses that saturate over this time window (dashed curves in Fig. 1a), generating step responses that form the basis of the b-wave component of the response. The resulting fits were subtracted from the whole ERG to provide an estimate of the resultant b-waves at each intensity (derived P2, Fig. 1b). Note that this derivation suggests that the non-monotonic variation of the empirical b-wave peak amplitude with intensity (ERG curves in Fig. 1a, red arrow) derives from a monotonic increase of the derived P2 wave (Fig. 1b) summing with the saturating receptor potential (P3; dashed lines in Fig. 1a). Thus, the results of this model fit imply that both the inferred receptor potential (P3) and bipolar-cell response (P2) are non-linear, though in different ways.

A key feature of their analysis is that the underlying model P3 component of the flash response (the receptor potential, as derived for cat retina by Granit [5], and termed the PIII component) is much slower than the ERG b-wave, effectively operating as a graded step response for the early time period of the ERG analyzed here, when it runs into the saturation range at the higher intensities. A notable aspect of these flash ERG data is the late crossover of the waveforms after about 70 ms to a negative-going response comparable in amplitude with the early negative peak of the a-wave (Fig. 1a). Notice also that these late-phase responses show a roll-back towards the baseline for higher-intensity flashes (Fig. 1a, yellow arrow). These features will prove to be discriminative aspects for the present ERG model (see discussion of Figs. 5, 6).

Hood and Birch [6] followed up the analysis depicted in Fig. 1a, b with a full model of the a/b-wave complex, as depicted in Fig. 1c, d. The basic concept is of an impulse response generator following by a static compressive nonlinearity for the receptor potential (P3), summing electrically with the output of a second stage that takes the temporal derivative of the resulting P3 and puts it through a second static nonlinearity followed by a second-stage linear filter, yielding the appropriate dynamic nonlinear response at the output.

It is noteworthy that recordings from bipolar cells [2], horizontal cells [11] and ganglion cells [3] have been shown to have a differential form of coupling with respect to the photoreceptor input, as implemented both in the Hood and Birch model of Fig. 1 and the present model (see Methods). The net output of this dynamic model (Fig. 1d), although designed to match a different dataset, expresses several of the features of the dark-adapted ERG flash series, but has the following shortcomings as a model of the low intensity dataset of Fig. 1a:

1. The a-waves are too broad, with much larger amplitudes than the empirical data.
2. The b-wave peaks do not decrease in amplitude as intensity is reduced.
3. The model does not account for the negative crossover seen in the empirical responses at long durations (Fig. 1a), which is comparable in magnitude to the negative peak of the a-wave.
4. Other issues addressed in Results.

**Fig. 2:**
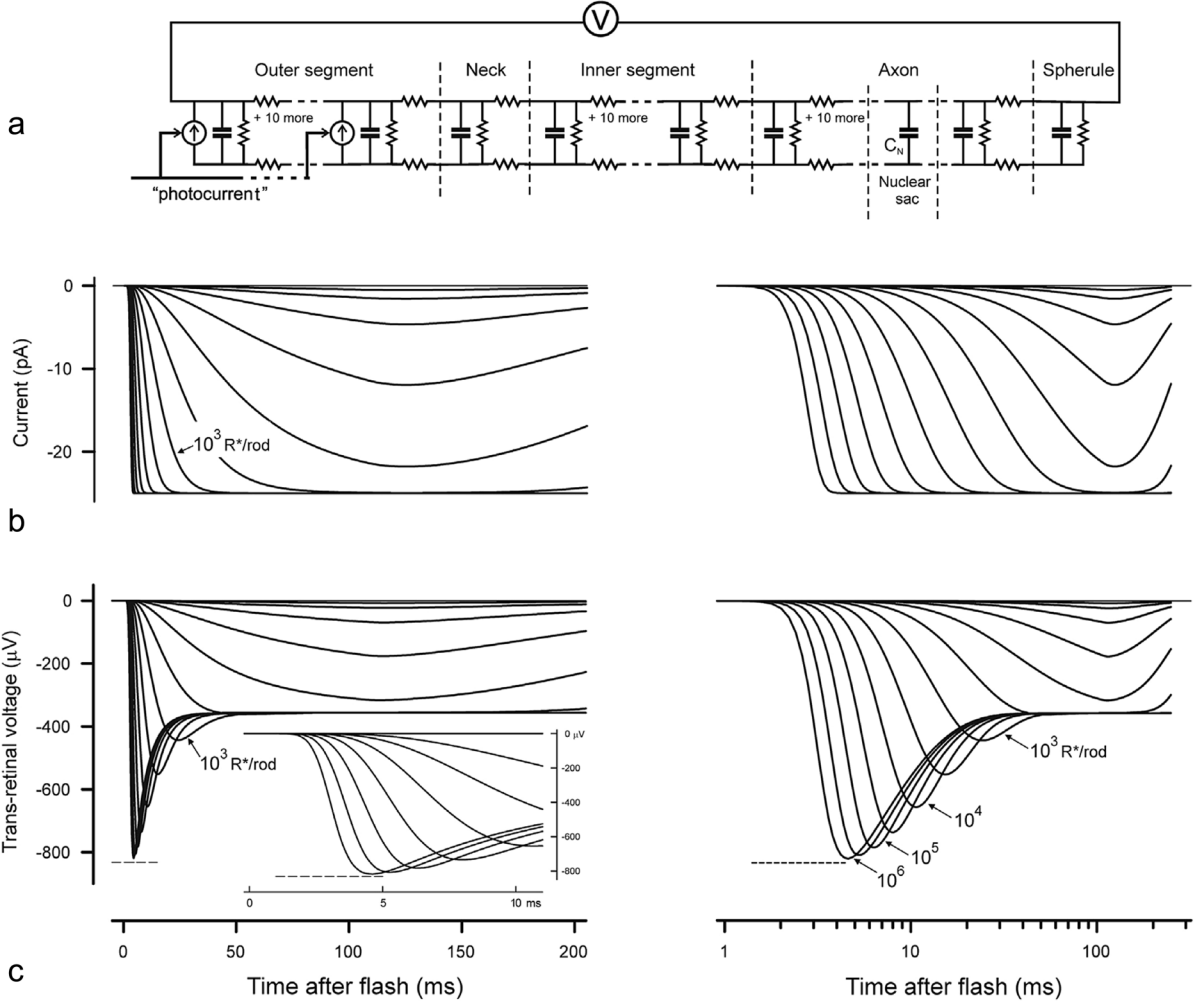
The Robson/Frishman SPICE (Simulation Program with Integrated Circuit Emphasis) model of the photoreceptor (P3) current and transretinal voltage responses. **a** Equivalent electrical circuit of a rod used to simulate trans-retinal voltage responses to outer-segment photocurrent. **b**, **c** Simulations of photocurrent and trans-retinal voltage responses to brief flash stimuli that give rise to 1 to 10.^6^ photoisomerizations/rod. Righthand sets of curves in **b** and **c** show the simulated voltage responses using log-time axes. As in Fig. 5 in Robson and Frishman [10]

A more recent modeling by Robson and Frishman focused on the rod outer segment contribution to the macaque transretinal ERG [10], their Fig. 5). They used a Hodgkin-Huxley style electronic ladder-network model (Fig. 2a) to capture the transretinal voltage dynamics, following the phototransduction cascade that determined the initial photocurrent (Fig. 2b). Their simulations reproduced the well-known behavior from single-cell recordings that the rod photocurrent has an impulse response peaking at about 120 *ms* (Fig. 2b), limited by an instantaneous saturating nonlinearity of the form derived empirically by Granit [5] for the P3 ERG component.

In contrast to the photocurrent behavior (Fig. 2b), the photovoltage responses that contribute to the overall ERG have the pronounced dynamic nonlinearity of a transient ‘nose’ for high-intensity flashes that relaxes to a saturating plateau (Fig. 2c), matching the recorded photoreceptor voltage responses (e.g., [8, 12]). This nose is attributable either to a voltage-dependent conductance [1], or a capacitive transient [10] in the outer nuclear layer of the retina. A key goal of the present modeling is to show the effects of this nose on the overall ERG structure recorded noninvasively from the human eye.

## Methods

The present simulations were programmed in Matlab based on the following equations:

1 $$ {\text{receptor }}\,{\text{potential}}\,{\text{ generator}}:\,R\left( t \right) = \left( {1 - k_{1} .{\text{log}}\left( I \right).\left( {t/\tau_{1} } \right).exp\left( {1 - t/\tau_{1} } \right)^{{n_{1} - 1}} } \right) $$

2 $$ {\text{capacitative}}\,{\text{ nose}}\,{\text{ generator}}:\,\,R^{\prime}\left( t \right) = R\left( t \right) + k_{2} .d\left( {R\left( t \right)} \right)/dt $$

3 $$ {\text{transient }}\,{\text{pulse}}\,{\text{ generator}}:\,T\left( t \right) = \left( {d\left( {R^{\prime}\left( t \right)} \right)/dt} \right) \otimes \left( {\left( {t/\tau_{2} } \right).exp\left( {1 - t/\tau_{2} } \right)^{{n_{2} - 1}} } \right) $$

4 $$ {\text{bipolar }}\,{\text{response}}\,{\text{ generator}}\,:\,B\left( t \right) = T\left( t \right) \otimes \left( {\left( {\frac{t - \Delta t}{{\tau_{3} }}} \right).\exp \left( {1 - \frac{t - \Delta t}{{\tau_{3} }}} \right)^{{n_{3} - 1}} .\left( {1 + I/n_{3}} \right)/I} \right) $$

5 $$ {\text{recording}}\,{\text{ filter }}\,{\text{function}}:\,f\left( t \right) = (\exp ( - t/\tau_{{{h}}} )) \, \otimes {\text{ exp}}({1 } - {\text{ t}}/\tau_{{{l}}} ) $$

6 $$ {\text{net }}\,{\text{waveform}}:\,ERG = R\left( t \right) + k_{3} .B\left( t \right) $$

where *t* is time, *I* is the flash intensity, *n*_1_, *n*_2_, *n*_3_, *n*_4_, are nonlinearity exponents, *k*_1_, *k*_2_, *k*_3_, τ_1_, τ_2_, τ_3_ are scaling constants, τ_*h*_, τ_*l*_ are the high- and low-pass time constants of the recording filter, and ⨂ denotes the convolution operator. The values of these constants are provided in the Appendix.

Note that the a-wave generator (Eq. 1) here is defined as a step response to light onset, which also serves as a model of the instantaneous flash response of the ERG under the assumptions of Hood and Birch [6] and others that the time constant of the photoreceptor P3 generator is much longer than the dynamics of the recorded ERG (as illustrated in Fig. 1). The transient component of this step response is implemented as an added transient in Eq. 2, as proposed by the Robson and Frishman [10] model of the nose as deriving from transient capacitive currents in the photoreceptors.

**Fig. 3:**
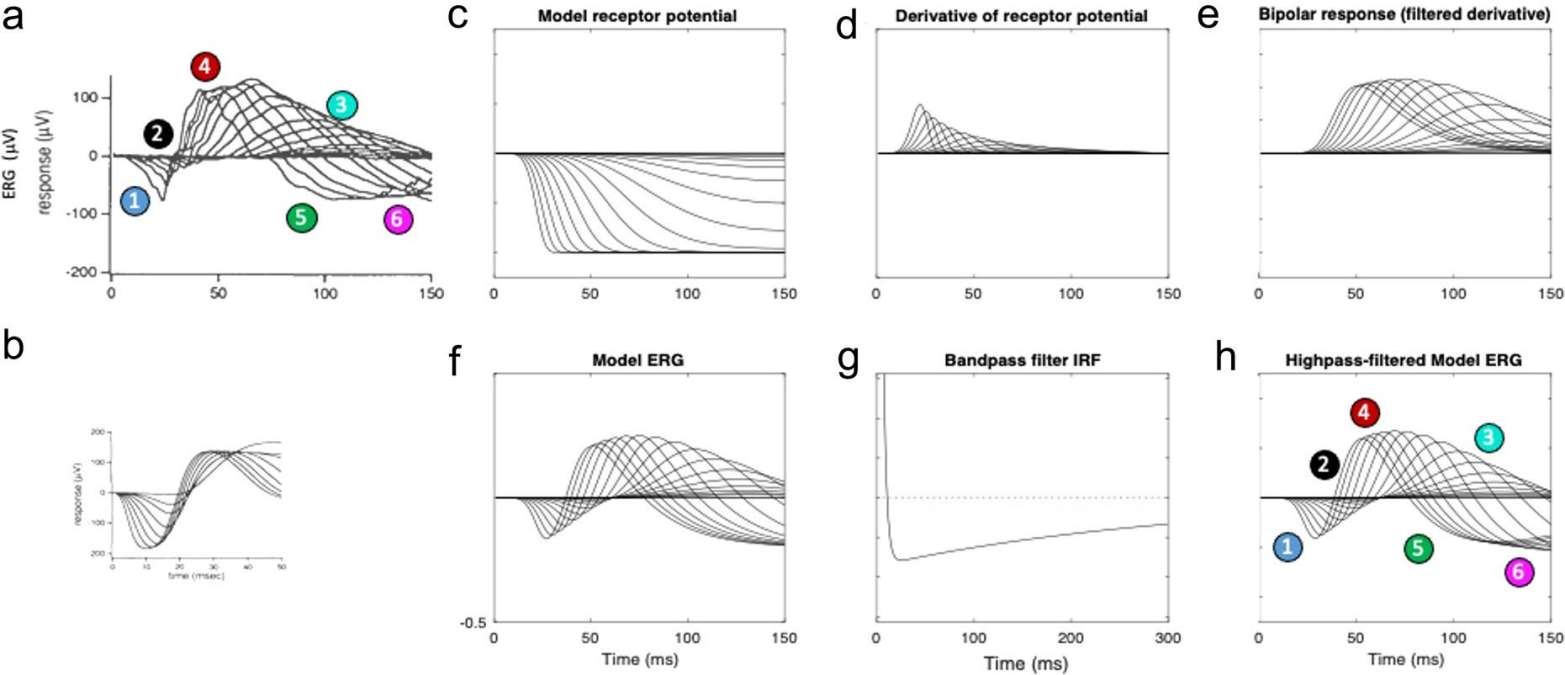
**a** Dark-adapted flash ERG responses from Hood and Birch [6]. For explanation of the numbered features, see text. **b** Model ERGs from [6] scaled to the same coordinates as for (**a**). **c**–**f** Present ERG model stages: simple photoreceptor potential (**c**), P2 transient pulse generator (**d**), monophasic P2 wave (**e**), and predicted DC-coupled ERG waveform (**f**). **g**–**h** Generation of the bandpass ERG prediction: **g**. bandpass filter impulse response function (IRF); **h**: bandpass filtered ERG prediction

**Fig. 4:**
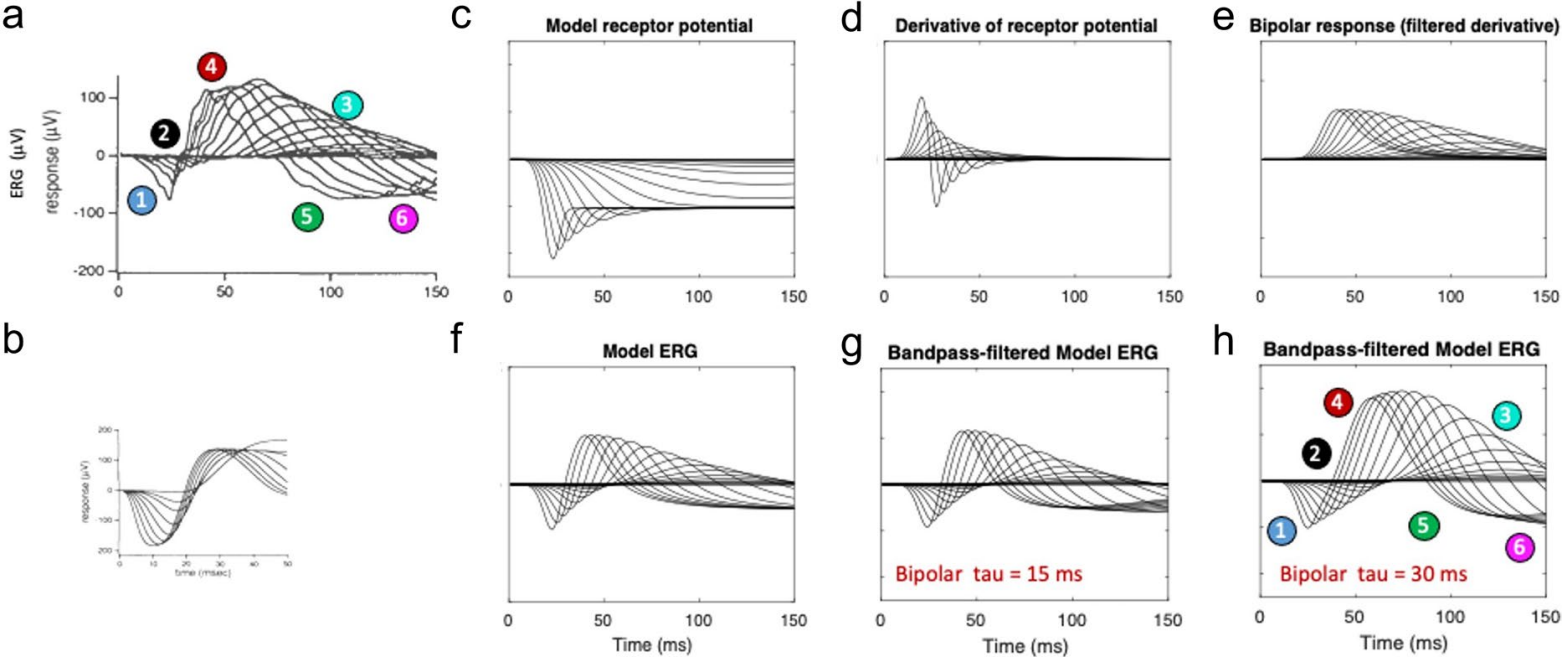
**a**–**f** Results, in the respective ERG model stages in the format of Fig. 3, of giving the photoreceptor potential a transient nose (**c**): biphasic P2 generator (**d**), slightly biphasic P2 waves (**d**), and predicted DC-coupled ERG waveform (**f**). **g** Resultant filtered waveform prediction showing that the nose shifts the balance between the a-wave and the late rebound. **h** Filtered waveform prediction with the b-wave time constant doubled from 15 to 30 *ms*

**Fig. 5:**
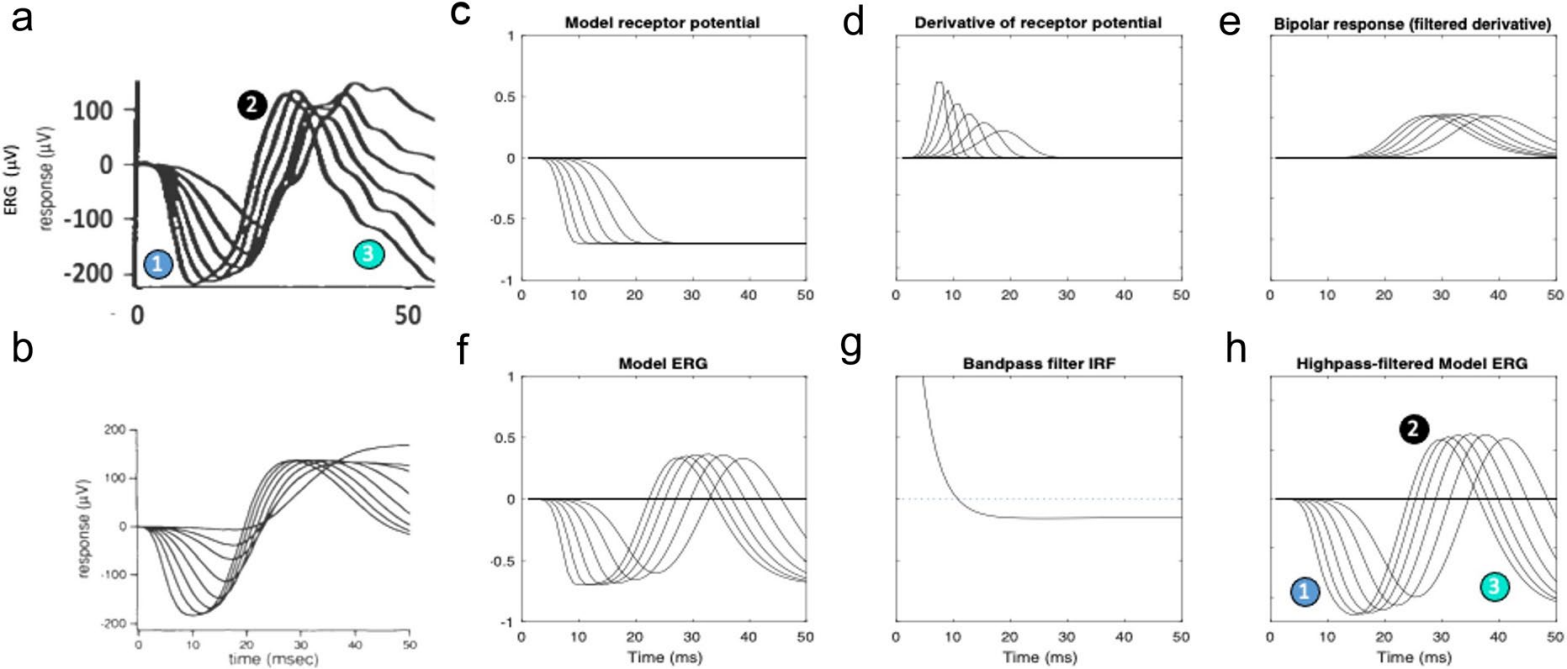
Match of the interim model to the Hood and Birch [6] high-intensity data shown in panel **a**, on their short time scale. **b** Hood and Birch model showing an adequate fit to the a-wave progression but major discrepancies from the b-wave properties. **c**–**h**. Extension of the new model in the format of Fig. 3 to the higher intensity range captures several key features of the data: (1) Broader a-wave; (2) narrow symmetrical peak for the b-wave; and (3) rebound back to strongly negative potential comparable with the a-wave peak. In the bandpass filtered waveforms (**h**), however, the filtering removes the sharp early peak evident in the a-waves of the unfiltered waveforms (**f**)

**Fig. 6:**
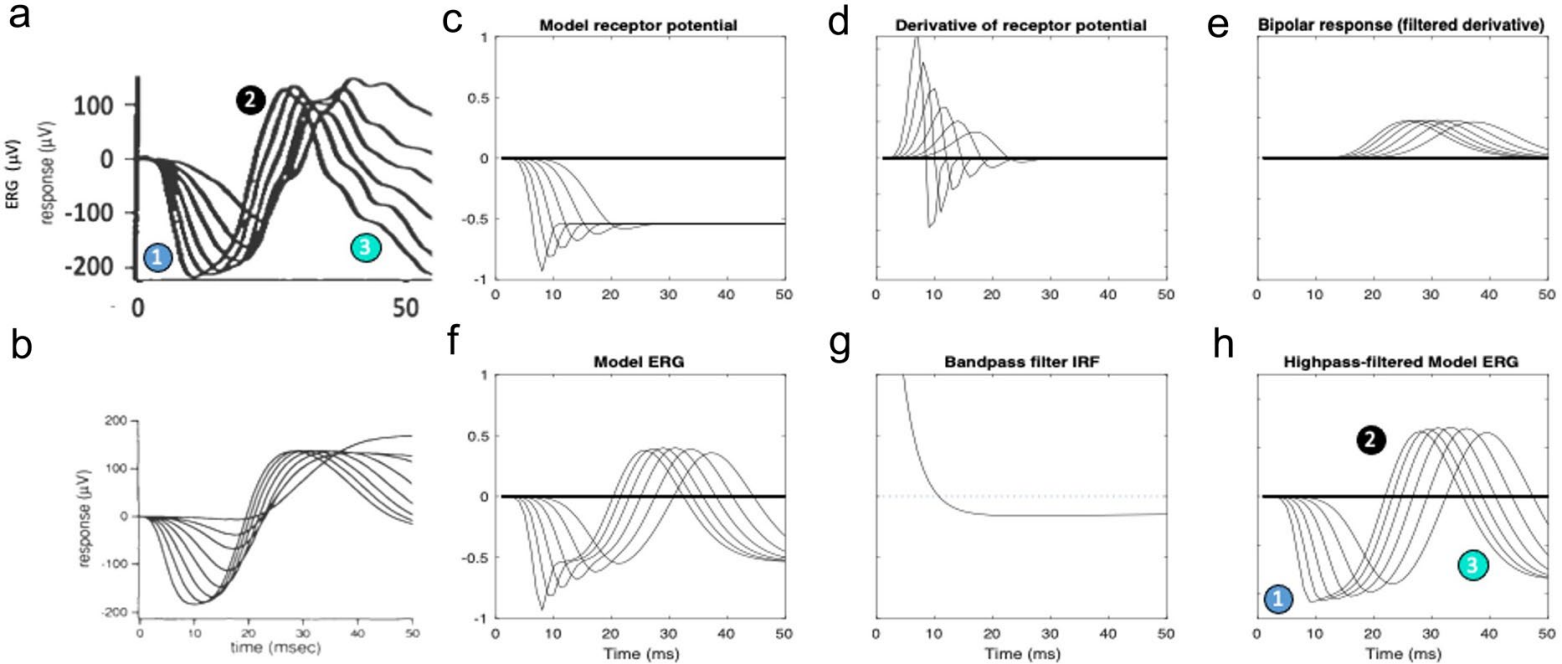
**a**, **b** The high-intensity ERGs (**a**) and Hood and Birch model (**b**) from Fig. 5. **c**–**h** the behavior of the model at the respective ERG model stages as in Fig. 5 after inclusion of the Robson and Frishman SPICE model for the photoreceptor potential. **c** Transient nose in the P2 waveform (Fig. 6c); **d** Biphasic P2 generator; **e** predicted b-wave after filtering the derivative of the receptor potential shown in (**d)**; **f** predicted DC-coupled ERG waveform; **g**: bandpass filter IRF; **h**: Resultant predicted (filtered) ERG waveform

According to the original publication of Hood and Birch, the ERG data were acquired as specified in Birch and Fish [4]. Specifically, the recording conditions were full-field ERGs obtained over a 4.1 log unit range of retinal illuminances (in approximately 0.2 log unit steps) with short-wavelength flashes from a strobe photostimulator (W47A; Xmax = 470 *nm*; half bandwidth = 55 *nm*) by a combination of calibrated neutral density filters and intensity settings on the photostimulator. Responses were obtained with a Burian-Allen bipolar contact lens electrode (Hansen Ophthalmic, Iowa City, IA) following pupil dilation (10% cyclopentolate hydrochloride and 1% phenylephrine hydrochloride) and 45 *min* of dark adaptation. Responses were amplified with a gain of 10,000 with bandpass filtering 3 dB down at 2 and 300 Hz (with one-pole filtering, David Birch, personal communication). For valid comparison, therefore, the present model was filtered in the same way (see Figs. 3, 4, 5 and 6).

## Results

1. The first analysis is to compare the results of the present simulation with the Hood and Birch low intensity flash ERG series (Fig. 3a) and with their simulation (Fig. 3b). Our initial model assumed a simple Granit [5] form of the receptor potential (Fig. 3c). The P2 generator (Fig. 3d) is modeled as a transient generated as the derivative of the P3 wave (Fig. 3d), such as from the transmission dynamics of the rod-bipolar synapse (as in the [6] model). The model bipolar response, or P2 (Fig. 3e) is then a temporally filtered version of the generator transient shown in Fig. 3d, and the net DC-coupled ERG prediction (Fig. 3f) is obtained as the sum of the model receptor potential (P3) and bipolar response (P2), (symbolized as *R*(*t*) and *B*(*t*) in Eq. 5, respectively). The predicted ERG (Fig. 3f) has a late crossover to a negative signal beginning at about 70–140 *ms*, depending on flash intensity, and remains strongly negative thereafter, as does the empirical ERG over this 150 *ms* time window. After applying the bandpass filtering used in the recordings (Fig. 3g), the final model (Fig. 3h) exhibits the late roll-back of the signal back toward the 0 µV baseline (feature 6 in Fig. 3a,h). This rollback is a function of the recording filter setting rather than being a feature of the DC-coupled ERG simulation. (Note that lack of access to the original data files of [6], precludes a direct overlay of the present model on those data, so the comparison is made by labeled features in the published data arrays.)

Thus, with the appropriate parametrization, this model (Fig. 3) captures almost all of the features of the empirical ERGs (Fig. 3a) not evident in the Hood and Birch [6] version: (1) the relatively short implicit times of the a-wave peaks, (2) the progressive reduction of the a- and b-wave time constants with intensity, (3) the increase of amplitude of the a- and b-waves with the logarithm of intensity (rather than a direct linear increase implied by the regular spacing in the mid-range of intensities, because the intensities were increased logarithmically), (4) the saturation of the b-wave amplitude increase at higher recording intensities (as opposed to the nearly uniform b-wave amplitude predicted by the Hood and Birch [6] model), (5) the crossover of the later response to a negative signal that reaches minima below that of the peak of the corresponding a-wave, and (6) the later roll-back of this late response towards baseline at the highest intensities.

2. The second issue is the effect of the initial transient in the P3 wave, as modeled by Robson and Frishman [10]. When the receptor potential in the present model is given a transient nose (Fig. 4c) corresponding to the voltage response of the Robson and Frishman SPICE model of Fig. 2c, the transient pulse generator becomes biphasic (Fig. 4d) and its effects carry through to the other stages of the retinal signaling. The manifestation of this early transient in the overall model ERG is the sharpening of the a-wave (Fig. 4f, h, feature 1), as seen in the empirical responses (Fig. 4a). With this parametrization, however, the balance between the a-wave and the late wave is no longer maintained: the crossover of the late responses to a negative signal never reach a minimum below that of the peak of the corresponding a-wave
3. Development of a no-nose high intensity model. In fact, the parametrization of the Hood and Birch model prediction was not intended to match the features of the low-intensity ERG series of Figs. 3a and 4a, but was designed to capture the high intensity ERG flash series modeled in their Figure 9 (from [4]), restricted to a 50 ms time window (reproduced here as Fig. 5a,b). (They did not provide information about their model performance at lower flash intensities.) Extension of the present model to the higher intensity range captures the key features that are missed by their model. The successful new features of the no-nose version of the present model at high intensity are: (1) a sharp corner in the a-wave (Fig. 5f); (2) a narrow symmetrical peak for the b-wave (Fig. 5h); and (3) a pronounced rebound back to strongly negative potential comparable with the a-wave peak (Fig. 5f, h). As seen in Fig. 5f, the DC coupled output of the new model captures all these of these features. However, the bandpass filtering (Fig. 5g) softens the sharp a-wave corner to the extent that it essentially disappears (Fig. 5h, feature 1).
4. The shortcomings of the waveform feature match of the no-nose version of the model (Fig. 5h) to the high-intensity data (Fig. 5a) when using the Granit form of the P2 generator as input (Fig. 5c) are rectified by the inclusion of a SPICE-model form of a P2 On-transient [10], as plotted in Fig. 6c. The effect of this On-transient carries through to provide a negative peak in the model ERG a-wave (Fig. 6f) that is softened by the bandpass filter to the requisite sharp corner (Fig. 6h, feature 1), matching the form of the high-intensity a-waves recorded by Birch and Fish [4] (Fig. 6a). The P2 onset transient thus provides a convincing explanation for this feature of the high-intensity recordings.

## Discussion

The present neuroanalytic model, though based on the same general principles as that of Hood and Birch [6] depicted in Figs. 1c,d and subsequent plots, provides a significantly better match than their own model to both their low and high flash-intensity series in respect to the six features enumerated in Figs. 3a and 4a. The new model is also compatible with the more recent modeling of the early stages of ERG generation by Robson and Frishman [10]. Note that, for the low-intensity series (Figs. 3, 4), the model replicates the feature in the data that the late crossovers for the smaller flash intensities become far more negative than their corresponding a-wave minima.

One of the key improvements in the present model is the way the intensity nonlinearity of the receptor potential is introduced as a gain control mechanism (Eq. 1) rather than as a static nonlinearity (panel C, Fig. 1). This approach provides a much better account of the empirical b-waves as a function of intensity (compare the present model output shown in Fig. 6h with the data in Fig. 6a and the Hood and Birch model output in Fig. 6b): (1) the shape of the b-wave across all intensities matches the data; (2) the decrease of the b-wave time-to-peak with intensity matches the data more closely; (3) the new model replicates the pronounced overshoot of the b-wave downslope to comparable negative values.

To capture the empirical reduction in b-wave amplitude at the highest intensities (feature 4 in Fig. 4a), a second compressive gain-control was introduced as the last expression in Eq. 4. This compressive function had to be located between the two convolution stages of the model in order to restrict the amplitude reduction to the P2 wave per se. Without it, the P2 amplitude would continue to rise in the manner of the P2 generator response of Figs. 3e and 4e, rather than saturating. The neural interpretation of this compression function could be a limit in the store of neurotransmitter molecules at the rod/bipolar synapse.

Another key difference from the model of Hood and Birch [6] is in the introduction of the On-transient ‘nose’ in the form of the receptor potential (Fig. 4c). This feature implements the empirical photovoltage responses recorded from primate rods and cones [8, 12] in the additive capacitive form suggested by Robson and Frishman [10]. The present analysis shows that the On-transient is primarily expressed in the character of the earlier portion of the a/b-wave complex. When the model receptor potential has no nose (Figs. 3c and 5c), the bandpass-filtered a-wave has a smooth, rounded character (Figs. 3h, 5h). However, the inclusion of a pronounced On-transient (Figs. 4c, 6c) sharpens the a-wave to manifest the corner (feature 1 in Fig. 6a, h) that is evident even in the bandpass-filtered Hood and Birch [6] data, implying that human dark-adapted receptor potentials have a moderate nose, as seen in primate rod responses (e.g., [8, 10, 12]).

In several respects, therefore, the present model offers a significant advance over previous models, accounting for several the various features of the dark-adapted ERG flash responses as a function of intensity for both the low- and the high-intensity regimes. Moreover, in the process of refining the model, including incorporation of components that reflect known photoreceptor electrophysiology (e.g., the ‘nose’ present in actual voltage recordings from photoreceptors; [8, 10, 12]), it identifies discriminative features that can facilitate physiologically relevant, quantitative incorporation of putative underlying neural contributions to the overall ERG. Since the ERG is a central ‘workhorse’ in clinical evaluation of retinal function, a tighter linkage of the model of the ERG to the underlying physiology and neural connectivity makes it correspondingly more valuable for improved characterization of both normal retinal mechanisms and their abnormalities in retinal diseases.

## Appendix

See Table

**Table 1:** Parameter values for Eqs. 1–6 generating the model outputs of Figs. 3, 4, 5 and 6

Dataset	Nose	*n*_1_	*n*_2_	*n*_3_	*n*_4_	*k*_1_	*k*_2_	*k*_3_	τ_1_	τ_2_	τ_3_	τ_*l*_	τ_*h*_
Low	0	8	8	1.4	3	2	0	0.65	150	15	8	2000	3.3
High	0	8	8	1.4	3	2	0	0.65	150	15	8	2000	3.3
Low	1	8	8	1.4	3	2	14	0.65	150	15	8	2000	3.3
High	1	8	8	1.4	3	2	14	0.65	150	30	8	2000	3.3

Time constants are in units of milliseconds

1.

## Declarations

### Statement of animal welfare

No animals were used in this study.
